# Significant Correlation Between the Infant Gut Microbiome and Rotavirus Vaccine Response in Rural Ghana

**DOI:** 10.1093/infdis/jiw518

**Published:** 2016-10-31

**Authors:** Vanessa C. Harris, George Armah, Susana Fuentes, Katri E. Korpela, Umesh Parashar, John C. Victor, Jacqueline Tate, Carolina de Weerth, Carlo Giaquinto, Willem Joost Wiersinga, Kristen D. C. Lewis, Willem M. de Vos

**Affiliations:** 1 Amsterdam Institute for Global Health and Development; 2 Center for Experimental and Molecular Medicine, Division of Infectious Diseases, Academic Medical Center, University of Amsterdam; 3 Laboratory of Microbiology, Wageningen University; 4 Behavioral Science Institute, Radboud University, Nijmegen, The Netherlands; 5 Noguchi Memorial Institute for Medical Research, College of Health Sciences, University of Ghana, Legon; 6 Department of Bacteriology and Immunology, and Immunobiology, University of Helsinki, Finland; 7 Division of Viral Diseases, National Center for Immunization and Respiratory Diseases, Center for Disease Control and Prevention, Atlanta, Georgia; 8 PATH, Vaccine Access and Delivery, Seattle, Washington; 9 Department of Paediatrics, University of Padova, Italy

**Keywords:** rotavirus vaccine, intestinal microbiome, mucosal immunity

## Abstract

**Background:**

Rotavirus (RV) is the leading cause of diarrhea-related death in children worldwide and 95% of RV-associated deaths occur in Africa and Asia where RV vaccines (RVVs) have lower efficacy. We hypothesize that differences in intestinal microbiome composition correlate with the decreased RVV efficacy observed in poor settings.

**Methods:**

We conducted a nested, case-control study comparing prevaccination, fecal microbiome compositions between 6-week old, matched RVV responders and nonresponders in rural Ghana. These infants' microbiomes were then compared with 154 age-matched, healthy Dutch infants' microbiomes, assumed to be RVV responders. Fecal microbiome analysis was performed in all groups using the Human Intestinal Tract Chip.

**Results:**

We analyzed findings in 78 Ghanaian infants, including 39 RVV responder and nonresponder pairs. The overall microbiome composition was significantly different between RVV responders and nonresponders (FDR, 0.12), and Ghanaian responders were more similar to Dutch infants than nonresponders (*P* = .002). RVV response correlated with an increased abundance of *Streptococcus bovis* and a decreased abundance of the Bacteroidetes phylum in comparisons between both Ghanaian RVV responders and nonresponders (FDR, 0.008 vs 0.003) and Dutch infants and Ghanaian nonresponders (FDR, 0.002 vs 0.009).

**Conclusions:**

The intestinal microbiome composition correlates significantly with RVV immunogenicity and may contribute to the diminished RVV immunogenicity observed in developing countries.

**(See the editorial commentary by Iturriza-Gómara and Cunliffe on pages 8–10.)**

Rotavirus (RV) is the leading cause of diarrhea-related death in children worldwide, with 95% of RV deaths occurring in low-income countries in Africa and Asia [1]. Oral RV vaccines (RVVs) have the potential to dramatically reduce the morbidity and mortality caused by RV infection, but RVVs demonstrate significantly lower efficacy in low-income countries [2]. Large clinical efficacy studies showed a combined vaccine efficacy against severe RV gastroenteritis ranging from 48% to 64% for both Rotarix and RotaTeq vaccines in Africa and Asia [3–5]. Emerging effectiveness data in Africa provides similar estimates of RVV protection [6]. This compares to an observed efficacy of 85%–98% against severe RV in trials in wealthier countries in Latin America and Europe [7–10].

Understanding the pathophysiologic mechanism driving this diminished efficacy in developing countries is critical, because even small improvements in vaccine efficacy could increase the number of children's lives saved by the vaccine by hundreds of thousands over the next 15 years [11]. There are several hypotheses as to why oral RVVs are underperforming in Africa and Asia [12]. These include interference with the first dose of coadministered oral poliovirus vaccine, RVV immune response suppression through high prevaccination levels of serum immunoblogulin (Ig) G, including transplacentally derived IgG, high levels of breast milk–derived RV-specific IgA, and HLA blood group antigen type [13–16]. However, none of these explanations have adequately and sufficiently explained the underperformance of RVV in developing countries, where vaccine efficacy can dip even lower than 50% in some settings. One underexplored hypothesis is that the intestinal microbiome may be modulating an infant's immune response to the enteric RVV [17]. We hypothesized that the composition of the intestinal microbiome is correlated with RVV response, that RVV responders have different intestinal microbes as compared with nonresponders and that these dissimilarities may contribute to the decreased efficacy of RVV found in resource-poor settings. To test these hypotheses, we conducted a nested, case-control study in Navrongo, Ghana, comparing the differences in intestinal microbiome composition and diversity between RVV seroconverters and nonseroconverters after vaccination with the Rotarix vaccine. We then compared these infants' microbiomes with those from a large group of age-matched healthy infant from the Netherlands, where RVV response is postulated to be high, similar to responses observed in other Northern European countries [10, 18].

## METHODS

### Study Design and Participants

#### Ghanaian Infants

The original trial within which this trial was nested was conducted in Navrongo, a rural setting in Northern Ghana where >70% of the population belong to the lowest wealth quintile in Ghana, in 2012. The neonatal and mortality rates are 24 and 46 deaths per 1000 live births, respectively [19].

All participating infants were healthy infants with a birth weight > 2000 g and/or a gestational age >38 weeks. The infants were enrolled at 6 weeks of age in a previously reported phase IV randomized clinical trial conducted in 2012 in Navrongo to evaluate the immunogenicity of the Rotarix vaccine after different dosing schedules (at age 6 and 10 weeks, 10 and 14 weeks, or 6, 10, and 14 weeks) (NCT01575197, clinicaltrials.gov) [20]. In this trial, all infants received concomitant standard Expanded Program on Immunization vaccinations, including the trivalent oral poliovirus vaccine and pentavalent vaccine (diphtheria, tetanus, whole-cell pertussis, hepatitis B, and *Haemophilus influenza* type).

Only infants from the 6- and 10-week and the 6-, 10-, and 14-week dose arms of the clinical trial were included in this microbiome study. A serum samples was collected before the receipt of the first dose of vaccine (at 6 weeks of age) and serum was collected again approximately 4 weeks after the last dose of vaccine (at age 14 or 18 weeks, depending on the study arm) for anti-RV IgA antibody measurements. Fecal samples were collected immediately before vaccination, at 6 weeks of age.

Infants were included in the study if during the original study their guardians had consented to additional testing of specimens in RV-vaccine related studies. Inclusion criteria further mandated that a baseline fecal sample was available and that there was no evidence of natural RV infection before vaccination (prevaccination IgA level ≥20 IU/mL). An IgA level ≥20 IU/mL was considered an indication of seroconversion and a surrogate marker for RVV protection against severe RV gastroenteritis [21].

Participating infants were then grouped as either RVV responders (postvaccination anti-RV IgA antibody ≥20 IU/mL) or RVV nonresponders (postvaccination anti-RV IgA antibody <20 IU/mL) and matched by hand in a 1:1 ratio using the following ranked variables: number and timing of doses of vaccine received (at age 6 and 10 weeks or 6, 10, and 14 weeks), sex, age at vaccination, RV season (defined as the date of the serum IgA level obtained 28 days after the last vaccination, between 1 December 2012 and 1 March 2013), ethnicity, height and weight at enrollment (including underweight, stunting, and wasting *Z* scores), and whether the infant regurgitated the vaccine after administration. The exact mode of delivery data and breastfeeding practices data are not known for the original Ghanaian study population. However, all infants in Ghana were delivered in the study hospital where >95% of deliveries are vaginal, and almost all infants delivered vaginally are breastfed. The nested trials were approved by the institutional review board of the Noguchi Memorial Institute for Medical Research and the research was conducted in accordance with good clinical practice guidelines.

#### Dutch Infants

In parallel, we compared the microbiome of both the Ghanaian responder and nonresponder infants with those in a cohort of healthy, age-matched Dutch control infants. The Dutch infants had not received RVV, but were assumed to be RVV responders, in line with ample clinical trial data demonstrating a >90% RVV seroconversion rate in Northern European countries [7, 10]. Fecal samples for these control infants were collected at approximately 30 days of age as part of a previously published study (the Bibo study) in which mothers and their children were followed up beginning with the third trimester of pregnancy [22, 23]. Pregnant women were recruited through midwife practices in Nijmegen and surrounding areas in the Netherlands. All parents provided written informed consent for participation in the study. The study was approved by the ethical committee of the faculty of social sciences at the Radboud University in Nijmegen. The infants' microbiomes were analyzed in the study using an identical protocol based on the Human Intestinal Tract Chip (HITChip) phylogenetic microarray [22]. Consent had been given for further use of the samples, which were entirely anonymous in our analysis.

### Laboratory Evaluations

#### Assays

Anti-RV IgA antibody was measured in serum using an enzyme-linked immunosorbent assay described elsewhere, with values expressed in international units per milliliter [24, 25].

#### Fecal Microbiome and Enteric Pathogen Analysis

In Ghana, fecal samples were collected by community health workers in infants' homes, transported in a cool box, and frozen to −20°C within 24–48 hours of collection. All samples were stored in 3% glycerol in frost-free freezers. Routine temperature monitoring did not indicate any freeze-thaw cycles. In the Netherlands, fecal samples were collected by parents at home and immediately stored at −20°C. The samples were transported in coolers with freezing cartridges or dry ice for further storage at −80°C. These procedures are both considered adequate for fecal microbiome analysis [26].

The fecal samples were analyzed by means of HITChip microarrays in duplicate, as described elsewhere [27]. In brief, total DNA was extracted from the fecal material by a repeated bead beating procedure using a modified protocol for the QiaAmp DNA MiniStool Kit (Qiagen), also as described elsewhere [28]. The 16S ribosomal RNA gene was amplified using primers that enabled incorporation of T7 promoter sequence at the 5-terminus of the amplicon. RNA was transcribed with amino-allyl modified nucleotides that were later coupled to cyanine (Cy) 3 or Cy5 dyes. Labeled RNA was fragmented, and 2 samples, each carrying a different dye, were hybridized in duplicate to the HITChip microarrays.

The HITChip microarray is a comprehensive and highly reproducible phylogenetic microarray that enables the parallel profiling and the semiquantitative analysis of >1100 phylotypes representing all major intestinal phyla grouped in 130 genuslike groups described for the human intestinal microbiome [27]. This high-throughput technique has been benchmarked with ultradeep pyrosequencing of 16S ribosomal RNA amplicons and next-generation parallel sequencing of intestinal metagenomes [29, 30]. After microarray hybridization, a sample was accepted only if 2 independent hybridizations (labeled with Cy5 and Cy3) correlated significantly (>95% Pearson correlation). This highly reproducible microbiome analysis allowed for direct comparison of all samples described in this study.

### Statistical Analysis

A χ^2^ test was used to determine statistically significant differences in baseline characteristics between Ghanaian RVV responders and nonresponders. The Shannon diversity index was used to measure the diversity of the microbiome per sample, including richness and evenness, using the hybridization signal of all probes included in the HITChip microarray [31]. Paired 2-tailed Student *t* tests were used to evaluate statistical significance.

Comprehensive multivariate statistical analyses were performed using Canoco 5.0 software for Windows [32]. Genus-level principal coordinate analysis and redundancy analyses were performed to evaluate differences in the overall microbial composition between the Ghanaian study groups (Ghanaian RVV responders and nonresponders). The 130 genuslike bacterial groups targeted by the HITChip microarray were used as biological variables, and the matching variables named above were used as background variables and visualized by means of inverse distance-weighted interpolation of the variable values over the component space. Monte Carlo permutation testing was used to assess the significance of the effect of these variables in the data set.

Generalized linear models were used to identify individual bacterial phyla and genuslike groups (class for Firmicutes) associated with RV seroconversion, as differing significantly either between Ghanaian nonresponders and responders or between the Ghanaian nonresponders and Dutch infants. *P* values corrected for the false discovery rate (FDR) were used to correct for multiple testing [33]. Bacterial taxa, whose log-transformed relative abundance was differed significantly (FDR-corrected *P* < .10) between the Ghanaian nonresponders and responders or between the Ghanaian nonresponders and Dutch infants, were considered correlated with RVV immunogenicity. These statistical analyses were performed with the program R [34]. To evaluate the similarity between Ghanaian infants (nonresponders and responders) and Dutch infants, we generated Pearson correlation scores and then performed an analysis of variance between Ghanaian nonresponders and responders.

## RESULTS

### Ghanaian Infants

A total of 234 infants (of which 74 were RVV responders) with prevaccination fecal samples were available from the original Ghana clinical trials and eligible for this study. A total of 52 RV responders were successfully matched to 52 nonresponders in a 1:1 ratio based on the predefined matching criteria. Of those, a total of 78 samples (equaling 39 per-protocol matched pairs) had sufficient DNA of good enough quality to be successfully analyzed using the HITChip pipeline.

No significant differences between the vaccine responders and nonresponders in Ghana were identified for any of the measured variables and baseline characteristics as described in Table 1. The diversity index (Shannon index) of the intestinal microbiome did not differ between infants who were RVV responders and those who were nonresponders (mean [SD], 4.40 [0.24] vs 4.41 [0.25], respectively; *P* = .87).

**Table 1.** Baseline Characteristics and Matching Characteristics of the 78 Infants Enrolled in the Nested Study in Ghana and Differences Between RVV Nonresponders and Responders as Determined With χ^2^ Tests

	Infants, No. (%)^a^		
Characteristic	RVV Nonresponders	RVV Responders	*P* Value
Total	39/78 (50)	39/78 (50)	…
Sex			>.99
Male	14/39 (36)	14/39 (36)	
Female	25/39 (64)	25/39 (64)	
Ethnicity			>.99
Nankam	15/39 (38)	15/39 (38)	
Kassem	24/39 (62)	24/39 (62)	
Age at vaccination mean (SD), d	42 (0.47)	43 (1.44)	>.99
Vaccination schedule			>.99
Arm 2: age 6 and 10 wk	12/39 (31)	12/39 (31)	
Arm 3: age 6, 10, and 14 wk	27/39 (69)	27/39 (69)	
RV season			.36
Yes	31/39 (79)	34/39 (87)	
No	8/39 (21)	5/39 (13)	
Malnutrition			.95
Underweight	1/39 (3)	0/39 (0)	.31
Stunting	2/39 (5)	3/39 (8)	.64
Wasting	1/39 (3)	1/39 (3)	>.99
Wasting	1/39 (3)	1/39 (3)	>.99

Abbreviations: RV, rotavirus; RVV, RV vaccine.

^a^ Data represent No. (%) of infants unless otherwise specified.

**Figure 1.**
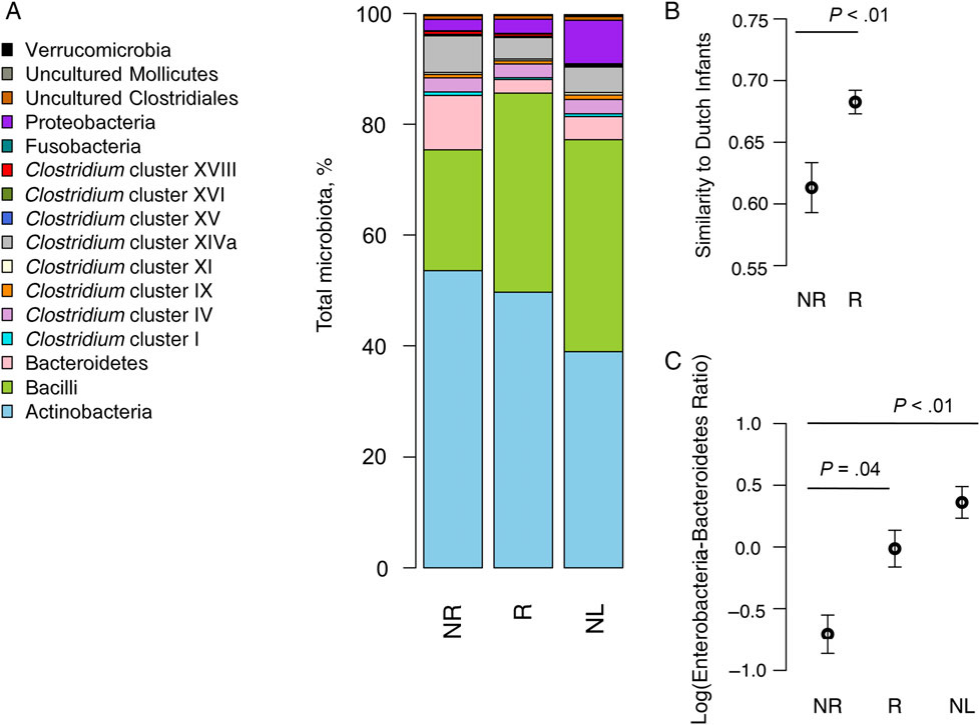
Overall microbiome composition. *A,* Bar plot of the percentage of each phylum in the total microbiome composition for the Ghanaian rotavirus vaccine (RVV) nonresponders (NR) and responders (R) and Dutch infants (NL). *B*, Ghanaian RVV responders are significantly more similar to Dutch infants than nonresponders. Y-axis shows mean similarity score as calculated by means of Pearson correlation. *C*, Ghanaian RVV responders and Dutch infants have a significantly higher enterobacteria-Bacteroidetes ratio than Ghanaian nonresponders. Y-axis shows the log-transformed ratio of all species of enterobacteria to all species of Bacteroidetes.

**Figure 2.**
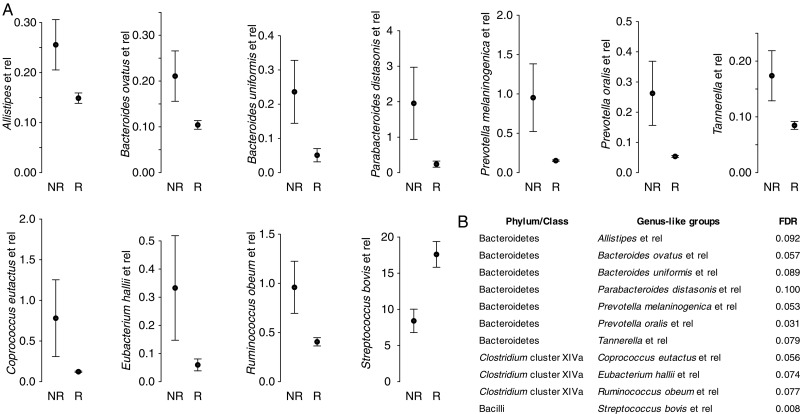
*A*, Comparison of the relative abundance (median signal intensity) of all bacterial genuslike groups with a statistically significant (false discovery rate [FDR] <0.1) difference in abundance between Ghanaian rotavirus vaccine (RVV) responders (R) and nonresponders (NR). Plots show means with standard errors. *B*, The FDR for each of the significantly different genera.

In Ghana, a high abundance of the Bacteroidetes phylum (FDR, 0.003), particularly several bacteria related to *Bacteroides* and *Prevotella* species, were significantly correlated with a lack of RVV response (Figures 1 and 2)*.* Conversely, the Bacilli phylum (FDR, 0.027) correlated significantly with RVV response, specifically bacteria related to *Streptococcus bovis* (FDR = 0.008) (Figures 1 and 2).

We further evaluated the high abundance of bacteria in the Bacteroidetes phylum in nonresponder infants. Recent literature has suggested that many bacteria in the Bacteroidetes phylum have a less immunogenic lipopolysaccharide (LPS) than Enterobacteriacae, such as *Escherichia coli* [35]*.* We therefore calculated an enterobacteria-Bacteroidetes ratio to provide a rough estimate of the presence of toxigenic LPS in the microbiome and compared the ratios between all groups. The enterobacteria-Bacteroidetes ratio was significantly higher in the Ghanaian responder infants (*P* = .04) than in nonresponder infants (Figure 1*C*).

Spearman correlation analyses were performed to assess whether the actual titer of the IgA—as opposed to RV seroconversion as a binary variable (≥20 IU/mL)—was correlated with specific bacterial groups. The Spearman correlation results showed that 33 genuslike bacterial groups were significantly correlated with RVV response (defined as an FDR <0.200). As with the results obtained when using IgA as a binary variable, bacteria related to *Streptococcus bovis* (FDR, 0.070) were the only bacteria whose abundance were correlated significantly (FDR, <0.1) with increased RVV titers. All other bacterial groups were significantly associated with lower RVV titers, and findings in only 1 bacterial group (bacteria related to Xanthomonadaceae) differed from those of the statistical analysis using IgA as a binary variable. Therefore, the Spearman correlation analysis using absolute IgA titers both mirrored and confirmed the findings attained when evaluating IgA as a binary variable with 20 IU/mL as a cutoff point. (Supplementary Figure 1).

Finally, the genuslike principal coordinate analysis indicated that the main variable differentiating the microbiome composition between the Ghanaian responders and nonresponders was RVV seroconversion, as calculated with Monte Carlo permutation testing (*P* = .01; FDR 0.12). (Supplementary Figure 2). None of the other environmental variables that were tested correlated with significant microbiome differences.

### Ghanaian Infants Compared With Dutch Infants

The Ghanaian infant microbiome compositions were then compared with the fecal microbiomes of 154 healthy Dutch infants. Of these Dutch infants, 62% were still receiving breast milk at 4 weeks of age, 42% were female, and 93% had been delivered vaginally; their mean (SD) birth weight was 3619 (457) g. When the Dutch infants were compared with the Ghanaian infants, their overall microbiome composition was significantly more similar to that in the Ghanaian RVV responders than to that in nonresponders (*P* = .002; Figure 1*B*).

**Figure 3.**
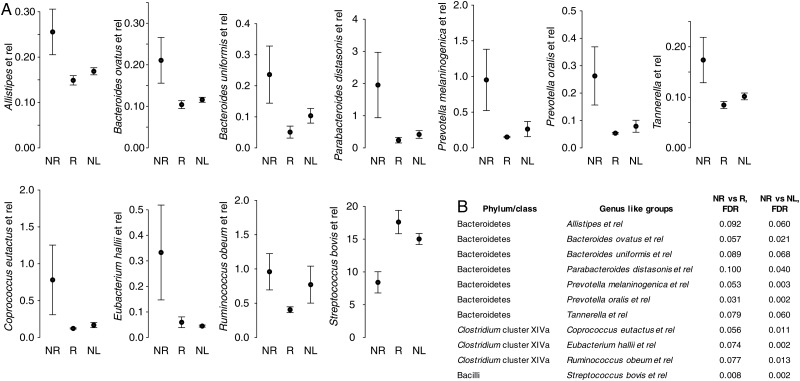
*A*, Comparison of the relative abundance (median signal intensity) of all bacterial genuslike groups with a statistically significant (false discovery rate [FDR], < 0.1) difference in abundance between *both* (1) Ghanaian rotavirus vaccine (RVV) responders (R) and nonresponders (NR) and (2) Dutch infants (NL) and Ghanaian RVV nonresponders (NR). Plots show means with standard errors. *B*, FDRs for the significantly different genera.

We then evaluated which phyla and bacterial genus groups differed significantly between Dutch infants and Ghanaian RVV nonresponders (see Supplementary Table 1) and subsequently assessed which of these bacterial groups also differed significantly in the comparison between Ghanaian RVV responders and nonresponders (Figure 3). At the phylum level, bacilli were significantly more abundant in both Ghanaian RVV responders and Dutch infants than in nonresponder infants (FDR for Dutch vs Ghanaian RVV nonresponders, 0.0002 ). The Bacteroidetes phylum was also significantly more abundant in Ghanaian nonresponders than both the Ghanaian responders and the Dutch infants (FDR, 0.006).

At the genus level, all bacterial groups that differed significantly between Ghanaian nonresponders and responders (FDR, <0.1) also differed significantly between Ghanaian nonresponders and Dutch infants. Bacteria related to *Streptococcus bovis* were more abundant in both Dutch infants and Ghanaian responders than in the Ghanaian infants without an RVV immune response (FDR, 0.0018). In parallel, several bacteria from the Bacteroidetes phylum (FDR, 0.002), particularly the *Bacteroides* and *Prevotella* genera, were more abundant in the Ghanaian nonresponders than in the Dutch infants (all Figure 3). Finally, the enterobacteria-Bacteroidetes ratio was also significantly higher (*P* < .01) in Dutch infants than in Ghanaian infants without an RVV immune response (Figure 1*C*).

## DISCUSSION

This study of Ghanaian infants demonstrates that the prevaccination intestinal microbiome differs significantly—on a genuslike, phylum, and overall composition level—between RVV responders and nonresponders in a rural, low-income setting in sub-Saharan Africa and that these microbiome differences are robustly recapitulated when Dutch infants are compared with RVV nonresponders.

RVV response was positively associated with the Bacilli phylum, specifically bacteria related to *Streptococcus bovis*. Several bacterial groups were significantly associated with a lack of RVV response—namely, bacteria belonging to the Bacteroidetes phlyum, especially several bacteria related to species from the *Bacteroides* and *Prevotella* genera.

The high abundance of Bacteroidetes in the microbiome of infants not responding to RVV is intriguing. Certain species from that phylum have been shown to express a form of LPS that is functionally and structurally different from the LPS expressed in some proteobacteria, such as *E. coli* [35, 36]. LPS is present in the outer membrane of most gram-negative bacteria and is a strong immunogenic stimulator of the innate immune system [37]. LPS structure varies from species to species, and variation—particularly in the lipid A core of LPS—can influence LPS' immunostimulatory capacity [38]. LPS derived from several Bacteroidetes species has recently been shown to have an impaired or even immune-inhibitory capacity to stimulate inflammatory cytokines in vitro when compared with LPS derived from *E. coli* [35].

Although our study could not measure infants' microbiome expression of a less toxigenic LPS, we chose to roughly evaluate this phenomenon by comparing the ratio of all enterobacteria to Bacteroidetes in each infant group. We did not compare specific enterobacteria or Bacteroidetes species because of some cross-hybridization at the species level in the HITChip microarray. This enterobacteria-Bacteroidetes ratio was significantly increased in both the Ghanaian RVV responders and Dutch infants compared with the nonresponders, raising the possibility that early microbiome colonization with bacteria expressing a less toxigenic or perhaps inhibitory form of LPS might be down-regulating innate immune responses to the live attenuated RV contained in the vaccine. Alternatively, more toxic LPS might be having an adjuvant effect on RVV responses in those Ghanaian infants capable of eliciting an immunogenic response to RVV.

Interestingly, in the Ghanaian responders, bacteria related to *Streptococcus bovis* was significantly correlated with RV response. The *Streptococcus bovis* group belongs to the *S. bovis–Streptococcus equinus* complex, which, like bacteria with toxigenic LPS, such as the *E. coli, Serratia,* and *Klebsiella* groups*,* can cross from being commensals to being opportunistic pathogens and whose cell surface proteins can trigger inflammatory responses [39]. Although speculative, these *S. bovis–S. equinus* complex bacteria could also be priming the immune system or acting as natural vaccine adjuvants.

An alternative hypothesis, given that the vaccines contain live attenuated virus, is that these bacteria might be expressing blood group antigens or glycans needed for RV replication, as has recently been demonstrated with norovirus and RV [40, 41]. Unfortunately, FUT2 secretor status, which is epidemiologically associated with RV gastroenteritis, is unknown among these infants.

This study has some limitations, restricting the strength of our findings. One of the most important is that, for the cohort of Dutch infants, we do not have RVV immunogenicity data and are predicting RVV response from randomized control studies showing >90% RVV efficacy in Europe [7, 10]. Because these were all healthy, at-term infants, we do predict that they would mount immune responses to RVV but are unable to demonstrate this. In addition, the study lacks specific data on breastfeeding and delivery practices for the infants in Ghana, and these may be confounders in our study results. Nevertheless, because >95% of deliveries are vaginal and all infants delivered vaginally are breastfed in this cohort, we do not expect mode of delivery and breastfeeding practices to be significant confounders in this study population. We also were not able to match our infants in Ghana for maternal antibodies to RV, such as breast milk anti-RV IgA and transplacentally acquired anti-G1 RV IgG antibody. High levels of maternally derived antibodies have been shown to diminish RVV immunogenicity, which could be contributing to the lack of response observed in our cohort and confounding the results [14, 15].

As another limitation, we used anti-RV IgA response as a correlate of vaccine protection, which can be an imperfect surrogate for vaccine efficacy [21]. Clinical vaccine efficacy would require larger sample sizes and follow-up. In addition, the intestinal microbiome is a complex ecosystem, and bacterial populations are in constant interplay with other intestinal inhabitants and pathogens, such as archae and eukaryotic microbes. This analysis of the microbiome did not evaluate the intestinal virome, fungiome, or parasites, and these could be immunologic mediators and influence bacterial populations. Finally, the associations between microbiome and RVV response are correlative and not causative and taken at a single time-point—directly before vaccination in a very young infants with high variability in their microbiome population. Nevertheless, our study used an identical, standardized, and reproducible technique to measure the intestinal microbiome in 2 geographic locations, and matching for numerous demographic variables in Ghana helps minimize possible study cofounders. As a consequence, our work is a springboard to understanding the mechanistic relationships between the intestinal microbiome and oral RVV responses in vulnerable infant populations and a call for more research to inform the design of evidence-based interventions to improve RVV efficacy worldwide.

## Supplementary Data

Supplementary materials are available at http://jid.oxfordjournals.org. Consisting of data provided by the author to benefit the reader, the posted materials are not copyedited and are the sole responsibility of the author, so questions or comments should be addressed to the author.

## Supplementary Material

Supplementary Data Table and Figure

Supplementary Figure
