# Supplementary material for: Significant Correlation Between the Infant Gut Microbiome and Rotavirus Vaccine Response in Rural Ghana
Source: J Infect Dis. 2016 Oct 31;215(1):34–41. doi: 10.1093/infdis/jiw518 (PMC5225256; doi:10.1093/infdis/jiw518)

**Supplementary Figure 1: (Left)** The genus-like bacterial groups with significant Spearman correlations in Ghanaian infants when evaluating the height of the post-vaccination IgA response. (**Middle**) Correlation index between the genus-like group and IgA height and heat map of the correlation: red is positively correlated with vaccine response and blue is negatively correlated with vaccine response. **+** indicates a significant correlation (p<0.05, FDR<0.2). **(Right):** Significance, expressed as adjusted-p level.

**Supplementary Figure 2**: Redundancy analysis (RDA) of matched pre-vaccination samples taken from 39 Ghanaian non-responders (red dots, NR) and 39 responders (green boxes, R) showing the 50 best fitting genus-like groups influencing the plot. The first and second ordination axes are plotted explaining 6.1 and 4% of the variability in the data set. The environmental variables rotavirus vaccine seroconversion is the only environmental variable influencing the plot as calculated by Monte Carlo Permutation Testing **(**p= 0.014, FDR= 0.12.

Supplementary Table 1: Statistically significant differences in the abundance of genus-like bacteria when comparing Ghanaian Non-responders with Dutch infants (FDR <0.1)

| **Phylum/Class** | **Genus-like groups** | **FDR** |
| --- | --- | --- |
| Actinobacteria | Eggerthella.lenta.et.rel. | 0.025 |
| Bacilli | Staphylococcus | <0.001 |
| Bacilli | Streptococcus.bovis.et.rel. | 0.002 |
| Bacilli | AerotolerantCocci | 0.002 |
| Bacilli | Streptococcus.mitis.et.rel. | 0.003 |
| Bacilli | Streptococcus.intermedius.et.rel. | 0.021 |
| Bacteroidetes | Prevotella.oralis.et.rel. | 0.002 |
| Bacteroidetes | Prevotella.melaninogenica.et.rel. | 0.003 |
| Bacteroidetes | Bacteroides.ovatus.et.rel. | 0.021 |
| Bacteroidetes | Parabacteroides.distasonis.et.rel. | 0.040 |
| Bacteroidetes | Bacteroides.plebeius.et.rel. | 0.040 |
| Bacteroidetes | Uncultured.Bacteroidetes | 0.046 |
| Bacteroidetes | Allistipes.et.rel. | 0.060 |
| Bacteroidetes | Tannerella.et.rel. | 0.060 |
| Bacteroidetes | Bacteroides.intestinalis.et.rel. | 0.064 |
| Bacteroidetes | Bacteroides.uniformis.et.rel. | 0.068 |
| Bacteroidetes | Bacteroides.fragilis.et.rel. | 0.079 |
| Clostridium cluster IX | Mitsuokella.multiacida.et.rel. | 0.068 |
| Clostridium cluster XI | Peptostreptococcus.anaerobius.et.rel. | 0.025 |
| Clostridium cluster XIV | Ruminococcus.gnavus.et.rel. | 0.011 |
| Clostridium cluster XIVa | Eubacterium.hallii.et.rel. | 0.002 |
| Clostridium cluster XIVa | Coprococcus.eutactus.et.rel. | 0.011 |
| Clostridium cluster XIVa | Ruminococcus.obeum.et.rel. | 0.013 |
| Clostridium cluster XIVa | Enterobacteria | 0.040 |
| Clostridium cluster XIVa | Clostridium.symbiosum.et.rel. | 0.042 |
| Clostridium cluster XIVa | Dorea.formicigenerans.et.rel. | 0.042 |
| Proteobacteria | Alcaligenes.faecalis.et.rel. | 0.011 |
| Proteobacteria | Campylobacter | 0.042 |
| Proteobacteria | Enterobacter.aerogenes.et.rel. | 0.060 |


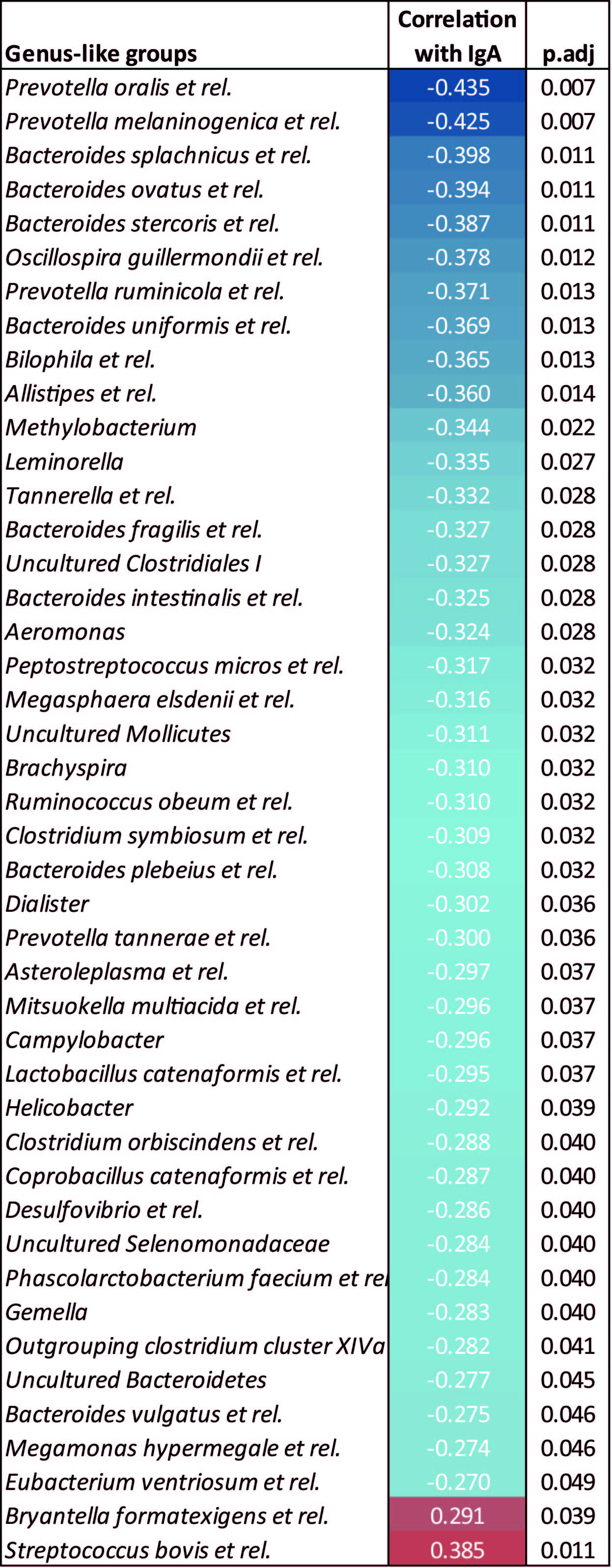

Supplement: Supplementary Data Table and Figure [file jiw518supp1.docx]
