# Supplementary figures and images for: Significant Correlation Between the Infant Gut Microbiome and Rotavirus Vaccine Response in Rural Ghana
Source: J Infect Dis. 2016 Oct 31;215(1):34–41. doi: 10.1093/infdis/jiw518 (PMC5225256; doi:10.1093/infdis/jiw518)

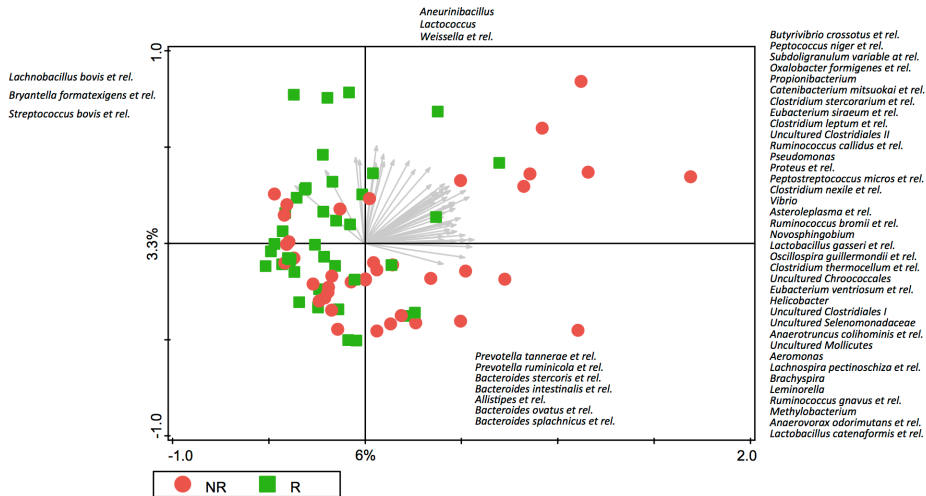

Supplement: Supplementary Figure [file jiw518supp2.pdf]
